# Calcium Homeostasis in Ventricular Myocytes of Diabetic Cardiomyopathy

**DOI:** 10.1155/2020/1942086

**Published:** 2020-11-13

**Authors:** Lina T. Al Kury

**Affiliations:** Department of Health Sciences, College of Natural and Health Sciences, Zayed University, Abu Dhabi 144534, UAE

## Abstract

Diabetes mellitus (DM) is a chronic metabolic disorder commonly characterized by high blood glucose levels, resulting from defects in insulin production or insulin resistance, or both. DM is a leading cause of mortality and morbidity worldwide, with diabetic cardiomyopathy as one of its main complications. It is well established that cardiovascular complications are common in both types of diabetes. Electrical and mechanical problems, resulting in cardiac contractile dysfunction, are considered as the major complications present in diabetic hearts. Inevitably, disturbances in the mechanism(s) of Ca^2+^ signaling in diabetes have implications for cardiac myocyte contraction. Over the last decade, significant progress has been made in outlining the mechanisms responsible for the diminished cardiac contractile function in diabetes using different animal models of type I diabetes mellitus (TIDM) and type II diabetes mellitus (TIIDM). The aim of this review is to evaluate our current understanding of the disturbances of Ca^2+^ transport and the role of main cardiac proteins involved in Ca^2+^ homeostasis in the diabetic rat ventricular cardiomyocytes. Exploring the molecular mechanism(s) of altered Ca^2+^ signaling in diabetes will provide an insight for the identification of novel therapeutic approaches to improve the heart function in diabetic patients.

## 1. Introduction

Diabetes mellitus (DM) is a chronic metabolic disorder commonly characterized by abnormally high blood glucose levels, resulting from defects in insulin production or insulin resistance, or both. Over the years, prevalence of diabetes has increased globally, and it is classified as one of the leading causes of mortality and morbidity. TIDM is characterized by decreased insulin secretion due to the damage in *β* cells of the pancreas [1, 2]. In contrast, TIIDM is characterized by decreased peripheral resistance to insulin, resulting in reduced insulin sensitivity to the skeletal muscles, adipose tissues, and liver [1, 3]. Hyperglycemia plays an important role in the onset and development of diabetes complications, mainly by generating reactive oxygen species (ROS) which causes lipid peroxidation and membrane damage. Furthermore, hyperglycemia results in excessive nonenzymatic glycation of proteins and formation of advanced glycation end products (AGE). The glycation modifications can further deteriorate the pathology of diabetes [4, 5].

Diabetic cardiomyopathy is one of the complications in DM. Electrical and mechanical problems, resulting in cardiac contractile dysfunction, are the major complications present in diabetic hearts. Clinical and preclinical studies have demonstrated a variety of diastolic and systolic dysfunctions in diabetic patients with the severity of abnormalities depending on the patients' age and duration of diabetes. Cardiac contractility is controlled through the precise interplay between several cellular Ca^2+^ transport protein complexes. During the excitation-contraction coupling process, the arrival of an action potential (AP) at a cardiac myocyte depolarizes the cell membrane leading to the opening of L-type Ca^2+^ channels and the influx of small amounts of Ca^2+^. This influx of Ca^2+^ triggers a much larger Ca^2+^ release from the sarcoplasmic reticulum (SR) via the ryanodine receptors (RyRs) and a transient increase in intracellular Ca^2+^ (Ca^2+^ transient). Ca^2+^ binds to troponin C and initiates and regulates the process of myocyte contraction. Myocyte relaxation takes place by the Ca^2+^ removal from the cytosol via main pathways including the uptake of Ca^2+^ into the SR through the SR Ca^2+^-ATPase (SERCA pump), transport outside the cell mainly via the Na^+^/Ca^2+^ exchanger (NCX), in addition to the plasma membrane Ca^2+^-ATPase (PMCA) [6]. A fourth pathway of the Ca^2+^ extrusion potentially involves mitochondria which are equipped with an efficient machinery for Ca^2+^ transport and are capable of storing large amounts of Ca^2+^ [7–10].

Disturbances in the mechanism(s) of Ca^2+^ signaling predictably have implications for cardiac myocyte contraction. It is well established that cardiovascular complications are common in both types of diabetes. Over the last decade, significant progress has been made in outlining the mechanisms responsible for the diminished cardiac contractile function in diabetes using different animal models of TIDM and TIIDM. The aim of this review is to evaluate our current understanding of the disturbances of Ca^2+^ transport and the role of main cardiac proteins involved in Ca^2+^ homeostasis in the diabetic rat ventricular cardiomyocytes. Exploring the molecular mechanism(s) of altered Ca^2+^ signaling in diabetes will provide an insight for the identification of novel therapeutic approaches to improve heart function in diabetic patients.

## 2. L-Type Ca^2+^ Channel

The cardiac voltage-gated L-type Ca^2+^ channel, Ca*_v_*1.2, is the main pathway for the Ca^2+^ entry into the cardiac cell. The fully functional Ca*_v_*1.2 channel is a heterotetrameric polypeptide complex containing the pore-forming Ca*_v_α*1c subunit, in addition to the accessory subunits Ca*_v_β*, Ca*_v_α*2*δ*, and Ca*_v_γ* [11]. The pore-forming Ca*_v_α*1c subunit contains the main biophysical and pharmacological properties of the channel and plays a critical role in excitation–contraction coupling. Entry of Ca^2+^ through Ca*_v_*1.2 channels shapes the plateau phase of the ventricular action potential and determines the action potential duration. In addition to the ion channel pore, the Ca*_v_α*1c subunit also consists of the voltage sensor, selectivity filter, and the determinants for the binding of drugs and toxins. The current through the Ca*_v_α*1c subunit is modulated by the interactions with the accessory subunits that are tightly bound to the Ca*_v_α*1c subunit. All of these accessory subunits play important roles in the regulation of both the biophysical properties and trafficking of L-type Ca^2+^ channels [11–13].

Compared to the surface sarcolemma, L-type Ca^2+^ channels are more localized in the T-tubule [13]. Within the T-tubule, most of L-type Ca^2+^ channels are concentrated in a specific region called dyad. Each dyad consists of clusters of L-Type Ca^2+^ channels on the sarcolemma closely opposed to clusters of RyRs on the SR membrane [6]. The two molecules are separated by a very limited space (10–15 nm) that enables a few Ca^2+^ ions to pass through the L-type Ca^2+^ channels and activate the RyRs. Such distribution forms the structural basis of excitation–contraction coupling [6, 13].

The L-type Ca^2+^channel activity is positively regulated by protein kinase A (PKA) phosphorylation. *β*-Adrenergic stimulation and the resulting PKA-mediated phosphorylation of key residues cause an approximately threefold surge in the L-type Ca^2+^ channel activity as a result of an increase in the channel open probability (*P*_*o*_) [14, 15]. The L-type current inactivates via two distinct mechanisms: a voltage-dependent inactivation, that is regulated by Ca_V_*β*, and a Ca^2+^-dependent inactivation, that is regulated by calmodulin (CaM). Both processes are thought to limit the amount of Ca^2+^ influx during the AP [16].

### 2.1. L-Type Ca^2+^ Channel in Type I Diabetes Mellitus

Various animal models are used to study TIDM. Deficiency in insulin production is achieved by a variety of mechanisms, ranging from chemical induction of beta cell damage (STZ-induced and alloxan-induced diabetes) [17] to genetic induction (e.g., AKITA mice) [18]. Previous studies in TIDM animal models have variously reported either no change [19–22] or reduction in the L-type Ca^2+^ current [20, 23–28] in ventricular myocytes isolated from the STZ-induced diabetic rat. For example, Chattou et al. 1999 found that, in rat diabetic myocytes, the density of the Ca^2+^ current was significantly reduced by TIDM in the range of test potentials between -10 and +50 mV. In addition, the fast time constant of the Ca^2+^ current inactivation was significantly higher in diabetic compared to normal myocytes which indicates that SR Ca^2+^ release-induced inactivation is delayed in TIDM. The decrease in the L-type Ca^2+^ current, which is the trigger for Ca^2+^-induced Ca^2+^ release (CICR) from SR, may explain the significantly lowered peak systolic intracellular Ca^2+^ in diabetic ventricular myocytes [20, 23–26]. Supporting this finding, Bracken et al. (2006) have shown that TIDM induced voltage-dependent decrease in contraction that was associated with the reduced L-type Ca^2+^ channel activity [28]. In cardiac myocytes of type 1 diabetic Akita Mice, decreased contractility was associated with reduced PI 3-kinase signaling and reduced cell surface expression of L-type Ca^2+^ channels. This change results in the decrease of the L-type Ca^2+^ current density that was reversed to control levels by insulin treatment and intracellular infusion of PI 3,4,5-trisphosphate [PI(3,4,5)P3] [27].

In contrast to the above findings, a recent study conducted by Smail et al. (2016) has shown that the L-type Ca^2+^ channel activation, inactivation, and recovery from inactivation were not significantly altered in epicardial and endocardial myocytes from STZ-treated rats [19].

### 2.2. L-Type Ca^2+^ Channel in Type II Diabetes Mellitus

In db/db obese type II diabetic mice, the depressed cardiac function was associated with reduction in the membrane permeability to Ca^2+^. Although the macroscopic L-type Ca^2+^ current was reduced in db/db cardiomyocytes, the single Ca^2+^ channel activity was similar, suggesting that diabetic myocytes express fewer functional Ca^2+^ channels [29]. The diminished T-tubular density was also observed in db/db mice in cardiomyocytes from mice with type II diabetes (db/db) [30]. Zucker diabetic fatty rat is a genetic model in which the male homozygous (FA/FA) animals develop obesity and TIIDM. In this model, earlier study has shown that the L-type Ca^2+^ current was reduced, and inactivation was prolonged over a range of test potentials in diabetic ventricular myocytes. Upregulation of the gene encoding the *α*1 subunit of the Cav1.2 ion channel (Cacna1c) may provide an early compensatory mechanism for the reduced density and prolonged inactivation of the L-type Ca^2+^ current demonstrated in myocytes from Zucker diabetic fatty rat compared to their respective controls [31]. In contrast to these findings, recent studies on the Goto–Kakizaki (GK) rat, a nonobese genetic model of TIIDM, have shown no change in the L-type Ca^2+^ channel activity in ventricular myocytes [32, 33]. Effects of TIDM and TIIDM on the L-type Ca^2+^ channel are summarized in Table 1.

## 3. The Ryanodine Receptor Type 2

Ryanodine receptor type 2 (RyR2) is a member of the RyR family. It is a macromolecular homotetrameric protein complex that regulates Ca^2+^ release from the SR during the process of excitation-contraction coupling in the heart. Sarcolemmal depolarization results in the entry of a small amount of Ca^2+^ to the cardiac cell. This influx of Ca^2+^ stimulates a large release of Ca^2+^ from the SR via RyR2 resulting in a transient rise of cytosolic Ca^2+^. In fact, activation of single RyR2 cluster (8–100 channels) results in an increase in the concentration of cytosolic Ca^2+^, known as a Ca^2+^ spark [34]. The summation of all Ca^2+^ sparks produced by activated RyR2 clusters throughout the cardiomyocyte leads to a Ca^2+^ transient that causes cardiac muscle contraction [35]. Recently, super-resolution imaging methods have provided an estimate for the number of RyRs in each cluster (dyad) from ≈14 in peripheral couplings to ≈100 in intracellular sites [34, 36]. A number of accessory proteins are associated with RyR2 and modulate its function including (1) the Ca^2+^ binding protein calmodulin which directly binds with and regulates RyR2 channels; (2) auxilliary proteins, calsequestrin, triadin, and junctin, which form the luminal Ca^2+^ sensor of RyR2 within the SR [37–39]; and (3) FK506 binding proteins (FKBP12 and FKBP12.6), which are believed to interact with RyR2 and stabilize the channel, preventing spontaneous Ca^2+^ release and SR Ca^2+^ leak [40]. In addition, the protein complex interacts with a number of enzymes including PKA, Ca^2+^/calmodulin-dependent protein kinase II (CaMK II), and phosphatases 1, 2A, and 2B that reversibly modulate the receptor phosphorylation state [41, 42].

### 3.1. The Ryanodine Receptor in Type I Diabetes Mellitus

To date, the molecular mechanism underlying RyR2 dysregulation during chronic diabetes is incompletely understood. Alteration in the sensitivity of RyR2 to the Ca^2+^ activation, oxidation of RyR2 by ROS and/or reactive carbonyl species [43–46], and functional uncoupling of RyR2 from L-type Ca^2+^ channels on the T-tubule membranes could be partly responsible for the dyssynchronous Ca^2+^ release from SR in diabetes [47].

In STZ-injected rats, earlier study conducted by Yu et al. (1994) reported a decrease in ^3^H-labeled ryanodine binding sites in diabetic myocardium, suggesting decreased density of the RyR protein [48]. Supporting this finding, Teshima et al. (2000) reported a decrease in the expression of RyR2 mRNA, 12 weeks after the STZ injection in the diabetic rat heart [22]. A more recent study also showed a significant decrease in the expression of RyR2 in 4-, 8-, and 12-week STZ-treated diabetic groups [49]. Together, the decreased density of RyR2 in the STZ rat heart can be explained by corresponding decrease in the mRNA expression.

It is well known that metabolic changes associated with diabetes increase the production of ROS. As the RyR structure is rich in free thiol groups, it is highly subject to oxidative stress, changing its tertiary structure and altering its sensitivity to Ca^2+^ [2, 50]. In an earlier study in 7-week sedentary type-1 diabetic rats, Ca^2+^ spark frequency was threefold higher, and evoked Ca^2+^ release was dyssynchronous with diastolic Ca^2+^ release. Although the steady state of the RyR2 protein (the state under which there is a continuous presence of critical Ca^2+^ to maintain the channel in its open state) was not altered, its response to Ca^2+^ was changed [51]. Yaras et al. (2005), however, found that in STZ-treated diabetic rats, Ca^2+^ transients exhibit significantly reduced amplitude and prolonged time courses, as well as depressed Ca^2+^ loading of SR. Spatiotemporal properties of the Ca^2+^ sparks were also significantly altered. Furthermore, protein levels of RyR2 were depleted [52]. Supporting these findings, the decreased expression of RyR2 receptors was reported earlier using the quantitative immunoblot technique. As a result, the decreased RyR function was responsible for the slow release of Ca^2+^ from SR and prolonged time to peak Ca^2+^ transients observed in diabetic rat myocytes [21]. Similar findings were also reported by other groups [51, 53, 54].

Alterations in the sensitivity of RyR2 to the Ca^2+^ activation could result from increased phosphorylation by PKA and CaMKII [43, 55, 56]. PKA was found to phosphorylate two sites of RyR2, primarily Ser2808 (in human and rodents) or Ser2809 (in rabbit) and Ser2030 (or Ser2031 in rabbit). CAMKII also phosphorylates the Ser2808 site, in addition to the Ser2814 (Ser2815 in rabbit) site [57]. The functional role of PKA and CaMKII-mediated phosphorylation of RyR2 has been implicated in many heart diseases, including heart failure [51, 53, 58]. For example, Marx et al. (2000) showed that PKA phosphorylation regulates the binding of FKBP12.6 to RyR2. PKA phosphorylation dissociates the regulatory subunit FKBP12.6 from the channel, resulting in the altered channel function which is manifested as increased probability of open state (*P_o_*), increased sensitivity to the Ca^2+^-induced activation, and destabilization of the channel [58].

In diabetic rat ventricular cardiomyocytes, Shao et al. (2009) showed that the RyR displayed about 1.5-fold increase in phosphorylation at Ser 2808 and Ser 2814 residues 7 weeks after STZ injection [51]. Interestingly, the PKA activity was reduced by 75%, but the CaMKII activity was increased by 50% [51]. Conversely, Yaras et al. (2005) reported that PKA-dependent phosphorylation of RyR2 was partly responsible for impaired intracellular Ca^2+^ signaling, as well as decreased SR Ca^2+^ load [52]. However, the role of CaMKII in phosphorylation of RyR2 and disturbance of Ca^2+^ signaling has been reported in STZ-diabetic rats [59] and *db/db* mice [30]. Interestingly, Tian et al. (2011) stated that the change in the RyR2 function observed in single channel recordings was independent of phosphorylation at either S2808 or S2814 sites. Instead, the increase in open channel probability (*P_o_*) and reduction in conductance were attributed to the increased responsiveness to cytoplasmic activators including Ca^2+^ [60].

### 3.2. The Ryanodine Receptor in Type II Diabetes Mellitus

In the prediabetic animal model of metabolic syndrome, the integrity of RyR2 was assessed by Ser 2809 phosphorylation, in addition to the receptor's ability to bind [^3^H]ryanodine. RyR2 phosphorylation at Ser 2809 was significantly elevated in the right and left ventricles from high-fat-fed dogs compared to normal controls. This hyperphosphorylation was associated with a decrease in RyR2 binding affinity in the right and left ventricles. However, there was no change in the level of expression of RyR2 [61]. In a more recent study on rats with metabolic syndrome, induced by a 16-week high-sucrose diet, cardiomyocytes exhibited altered Ca^2+^ signaling that was partly attributed to increased phosphorylation and altered RyR2 function [62]. Gaber et al. (2014) found that, in the GK rat model, changes in ventricular cardiomyocyte shortening and Ca^2+^ signaling were associated with a decrease in RyR2 mRNA levels [63]. Supporting this finding, prolonged SR Ca^2 +^ release and associated reduced RyR2 expression and increased phosphorylation were reported in the right atrial myocardium of TIIDM patients [64].

Diabetes and obesity are associated with an increased risk of arrhythmia and sudden cardiac death that could be partly attributed to abnormal lipid accumulation. Recently, the transgenic mouse model of cardiac lipid overload, with the cardiac-specific overexpression of peroxisome proliferator-activated receptor gamma (PPAR-*γ*), was used to study the change in Ca^2+^ handling [65, 66]. The PPAR-*γ* overexpression was found to perturb the intracellular Ca^2+^ homeostasis in cardiomyocytes leading to ventricular arrhythmias and cardiac sudden death in animals. The results of a recent study conducted by Joseph et al. (2016) showed that PPAR-*γ* cardiomyocytes had more frequent triggered activity, increased sparks, and SR Ca^2+^ leak. This was attributed to the significant increase in RyR2 oxidation [65]. Other studies have also reported that in vitro oxidation of RyR2 increases the channel response to cytoplasmic Ca^2+^ concentration and favors Ca^2+^ release in isolated cardiomyocytes, generating Ca^2+^ waves and arrhythmias [67, 68].

In mice fed with a high-fat diet (HFD), more frequent occurrence of arrhythmic episodes was associated with an enhanced response of single RyR2 channels to cytoplasmic Ca^2+^. At the molecular level, RyR2 channels from HFD-fed mice had substantially fewer free thiol residues, suggesting that redox modifications were responsible for the higher activity of RyR2 [69]. Effects of TIDM and TIIDM on RyR2 are summarized in Table 2.

## 4. The Sarcoplasmic Reticulum Ca^2+^-ATPase

SERCA pump plays a predominant role in cardiac excitation–contraction coupling and cardiac contractility. This pump is encoded by a family of three genes, SERCA1, 2, and 3, which are spliced in several isoforms. To date, more than 10 different SERCA isoforms have been identified at the protein level. In the cardiac tissue, SERCA2a is the predominant form which is responsible for facilitating the storage of Ca^2+^ in the SR. The function of the SERCA2a pump is modulated by the endogenous molecules phospholamban (PLB), sarcolipin (SLN), and by direct phosphorylation through CaMK II. In the dephosphorylated form, PLB inhibits SERCA2a, while PKA-dependent phosphorylation of the phosphoresidue serine-16 or Ca^2+^/calmodulin-dependent phosphorylation of threonine-17 reverses this inhibition [70, 71]. SERCA2a is also under the control of CaMK II, which has been shown to phosphorylate SERCA2a on residue serine-38 and enhance and Ca^2+^-reuptake into the SR [72]. These effects that are mediated through phosphorylation result in an overall increased SR Ca^2+^-load and enhanced contractility.

### 4.1. The Sarcoplasmic Reticulum Ca^2+^-ATPase in Type I Diabetes Mellitus

Because SERCA2 plays a major role in muscle contraction, various investigations have focused on understanding its role in cardiac disease. Many studies have reported that the SERCA2a expression and activity were decreased in a number of pathophysiological conditions including diabetes [73]. In TIDM, decreased activity of SERCA2a was associated with decreased level of mRNA levels or expression of protein, increased formation of ROS, change in the expression of PLB, and increased posttranslational modification such as increased carbonylation, glycation, and O-GlcNAcylation (Table 3). For example, in the STZ-induced diabetic rat heart, the expression of SERCA2a mRNA was significantly reduced 3 weeks after the STZ injection [22]. Kim et al. (2001) found that the maximal Ca^2+^ uptake and the affinity of SERCA2a for Ca^2+^ were decreased while the exogenous phosphorylation level of PLB was increased in STZ-induced diabetic rat SR. Levels of both mRNA and protein of PLB were significantly increased in the diabetic hearts, whereas those of SERCA2a were significantly decreased. Consequently, the relative PLB/Ca^2+^-ATPase ratio was 1.88 in the diabetic hearts, and these changes were correlated with changes in the rates of SR Ca^2+^ uptake [74]. Choi et al. (2002) also found that the depression in the SR function was associated with decreased SERCA2a and increased nonphosphorylated PLB proteins [21]. Supporting these findings, Bidasee et al. (2004) found that the hearts of 8-week STZ-treated animals expressed lower levels of the SERCA2a protein and higher levels of monomeric unphosphorylated PLB [75]. Decreased function of SERCA2a was also reported in other studies including studies on the alloxan model of TIDM [20, 76, 77].

Oxygen-derived free radicals have been reported to damage SERCA, leading to cellular Ca^2+^ overload. For example, earlier study by Okabe et al. (1983) showed that a decrease in Ca^2+^ uptake occurs in isolated SR after exposure to xanthine/xanthine oxidase, an enzymatic system capable of generating superoxide radicals [78]. Zu et al. (1997) found that hydroxyl radical inhibits the SERCA2a function by directly attacking the ATP binding site [79]. A more recent study by Ying et al. (2008) showed that exposure of cardiac SR membranes directly to peroxynitrite reduces the SERCA2a activity by oxidizing Cysteine 674, as well as interfering with the ATP binding site [80].

### 4.2. The Sarcoplasmic Reticulum Ca^2+^-ATPase in Type II Diabetes Mellitus

Previous studies in TIIDM animal models have variously reported either no change, decreased or increased expression of SERCA2a. Although Stølen et al. (2009) reported no change in the SERCA2a expression in the *db/db* mouse, the decreased activity of SERCA2a was attributed to the increased PLB expression [30]. Similarly, in ventricular myocytes isolated from adult rats fed on sucrose for 9-12 weeks, shortening/relengthening were significantly shorter compared to starch- (ST-) fed controls. Although the SERCA2a expression was unaltered, the inhibition was associated with decreased SR Ca^2+^ uptake and increased PLB phosphorylation [81].

In contrast to the above findings, both a decrease and an increase in the SERCA2a expression were observed in *Zucker Diabetic Fatty* rat, an early TIIDM model. While Young et al. (2002) reported a decrease in the SERCA2a expression and cardiac contractility [82], a more recent study conducted by Fredersdorf et al. (2012) showed that the SERCA2a expression is upregulated, whereas the expression of PLB mRNA was reduced. The changes were associated with a significant increase in SR Ca^2+^ uptake. Interestingly, the SERCA2a expression and SERCA/PLB ratio in diabetic animals were further increased by insulin treatment. From a pathophysiological point of view, insulin-induced upregulation of SERCA2a could be regarded as a feedback mechanism in handling the volume overload caused by high glucose levels in the early phase of TIIDM, when insulin levels are high [83]. Effects of TIDM and TIIDM on SERCA2a are summarized in Table 3.

## 5. The Sodium-Calcium Exchanger

The NCX is an electrogenic transporter located at the plasma membrane that catalyses the countertransport of Na^+^ and Ca^2+^. To date, 4 isoforms have been identified for NCX, namely, NCX1, NCX2, NCX3, and NCX4 [84]. The cardiac isoform, NCX1, is organized into ten transmembrane segments (TMSs) with a large cytoplasmic loop between TMSs 5 and 6 that plays a regulatory role. Ion transport is associated with two regions of intramolecular similarity named *α* repeats. They consist of TMSs 2–3 and TMSs 7–8 and their connecting links [85]. NCX1 plays an important role in Ca^2+^ homeostasis, typically by operating in forward mode to extrude one Ca^2+^ ion for 3 Na^+^ ions. The direction of Ca^2+^ transport reverses at membrane potentials near that of the AP plateau, generating an influx of Ca^2+^ into the cell [86]. NCX1 is regulated by intracellular Ca^2+^ [87], signaling lipid phosphatidylinositol-4,5-bisphosphate (PIP_2_) [86], and free radicals, as well as PKA and PKC [88, 89]. Alterations in the ionic and electrical conditions that accompany cardiac diseases promote reverse-mode of the NCX1 activity, leading to Ca^2+^ overload and electrical dysfunction [86].

### 5.1. The Sodium-Calcium Exchanger in Type I Diabetes Mellitus

Earlier studies have shown that the NCX current density was reduced [20, 26, 90, 91], and current inactivation was prolonged [26] in ventricular myocytes from STZ-induced diabetic rat. These variations in amplitude and kinetics of the current were accompanied with reduced NCX mRNA [90] and reduced or unaltered NCX protein in the STZ-induced diabetic rat heart [21, 92, 93]. In alloxan-injected rats, the NCX function was depressed 2 weeks after diabetes induction [94]. Most recent data from our lab has shown that the NCX current was significantly smaller in endocardial and epicardial ventricular cardiomyocytes compared to controls, 5-6 months after the induction of diabetes with STZ [95].

Despite the fact that all of the abov-mentioned studies have supported the decrease in NCX function in TIDM, results from the Akita(ins2) TIDM model showed an increase in the NCX expression as a compensatory mechanism in response to reduced contractility in the heart. Such increase was protective against systolic failure [96].

### 5.2. The Sodium-Calcium Exchanger in Type II Diabetes Mellitus

In mice with TIIDM (db/db), either no change or increased activity of NCX1 was observed. For example, in insulin-resistant sucrose-fed rats, the normal expression of NCX1 was observed [81]. Similarly, Ricci et al. (2006) found no change in the NCX current density in HFD mice [97]. However, Stølen et al. (2009) found an increased activity of NCX1in TIIDM (db/db) [30]. An increased NCX1 gene expression was observed in human with TIIDM and was associated with comparable left ventricular hypertrophy [98]. Effects of TIDM and TIIDM on NCX1 are summarized in Table 4.

## 6. Effect of Advanced Glycation Products on Ca^2+^ Handling Proteins in Diabetes

Chronic hyperglycemia results in excessive formation of advanced glycation end products (AGE). The glycation modifications can further deteriorate the pathology of diabetes [4, 5]. AGEs are a heterogenous group of molecules resulting from the nonenzymatic glycation and oxidation of proteins and lipids in the presence of reducing sugars. AGEs may alter cellular function through crosslinking of cellular proteins or by activating the AGE receptor (RAGE). In cardiomyocytes, AGEs were shown to crosslink the domains of both the RyR and SERCA2a [99]. Yan et al. (2014) showed that the AGE/RAGE signal enhanced Ca^2+^ spark-mediated SR Ca^2+^ leak, which resulted in partial depletion of the SR Ca^2+^ content and consequently, decreased systolic Ca^2+^ transient. Altogether, these effects have contributed to contractile dysfunction reported in diabetic cardiomyopathy [100, 101].

As mentioned earlier, the RyR2 structure is rich in free thiol groups and therefore, it is highly susceptible to oxidative stress. Hegab et al. (2017) found that the AGE-induced activation of RAGE enhanced the activity of NADPH oxidase and hence the production of ROS. This was accompanied with activation of p38 kinase, nuclear translocation of NF-*κ*B, and subsequently induction of inducible nitric oxide synthase (iNOS) expression, leading to increased NO production. Elevation of ROS and NO was found to alter Ca^2+^ handling through *S*-nitrosylation of key proteins such as SERCA2a, RyR2, and L-type Ca^2+^ channel [100, 102].

The relationship between diabetes-induced decrease in the RyR2 activity and the formation of AGE during chronic diabetes was also shown in other studies. Bidasee et al. (2003) have shown that AGEs are formed on RyR2 during diabetes. RyR2 from 8-week STZ-induced diabetic rat hearts contained several noncrosslinking AGEs. Noticeably, decreased ability to bind [^3^H]ryanodine and altered sensitivity to Ca^2+^ indicated the loss of functional integrity of RyR2 from these hearts [2]. In fact, formation of AGEs on RyR2 was not the only contributor to RyR2 dysfunction. In a previous study conducted by the same group on 6-week STZ-diabetic rat hearts, it was shown that the dysfunction of RyR2 stems in part from diabetes-induced increase in its disulfide bond content [103]. Furthermore, glycation of RyR2 was found to alter its gating properties and was associated with increased SR Ca^2+^ leak, elevated mitochondrial Ca^2+^ content, and concomitant mitochondrial Ca^2+^ overload and damage [104] .

SERCA2a is susceptible to posttranslational modifications during diabetes. It has been identified as a prominent target of glycative damage. Hearts from 8-week-old diabetic rats treated with STZ showed several cytosolic SERCA2a peptides, modified by single noncrosslinked and crosslinked AGEs. Lysine residues within the actuator domain (*A*, cytoplasmic) and phosphorylation domain (*P*, cytoplasmic) were crosslinked to arginine residues within the nucleotide binding domain (*N*, cytoplasmic) via pentosidine AGEs. 2 weeks of insulin treatment initiated after 6 weeks of diabetes significantly improved the cardiac function and also prevented the formation of crosslinking AGEs on SERCA2a. It is suggested that the disruption in the tertiary structure by AGE complexes prevented the structural movements required for translocating Ca^2+^ from the cytosol to the lumen of the SR and resulted in a decrease in the SERCA2a activity [75]. Other studies have identified carbonylation and O-GlcNAcylation as important mechanisms that contribute to the loss of the SERCA2a activity and diastolic dysfunction in a rat model of TIDM [46, 105].

## 7. Targeting Ca^2+^ Handling in Diabetes

Taken together, studies strongly suggest that several facets related to Ca^2+^ handling are dysregulated in diabetic cardiomyopathy, including altered expression and/or activity levels of the L-type Ca^2+^ channel activity, RyR2, SERCA2a, and NCX. Therefore, targeting these proteins provide potential therapeutic approaches to improve cardiac cell function in diabetes. Many studies have shown that the L-type Ca^2+^ channel activity is either unaltered or reduced in diabetes. The diminished Ca^2+^ entry through the L-type Ca^2+^ channel is a critical contributor to the negative effect on cardiac contractility observed in diabetic cardiomyopathy, and therefore, an increase of the trigger produced by the L-type Ca^2+^ current will increase the amplitude of Ca^2+^ transients and contraction. Gain-of-function mutations in the channel's *α*1-subunit or other proteins favoring cellular depolarization might be beneficial in diabetic cardiomyopathy. For example, mutations that increase the window current and maximal conductance for Ca^2+^ will augment the trigger for RyR2-mediated Ca^2+^ release, thereby improving the systolic function in the diabetic heart. Although the increased Ca^2+^ entry into the myocytes substantially contributes to the positive inotropic effect, it is worth noting that excess Ca^2+^ influx through the L-type Ca^2+^ channel is likely to contribute to intracellular Ca^2+^ overload.

The NCX function is also reduced according to many diabetic models. In fact, inhibition of NCX in the forward mode will further increase the cellular Ca^2+^ content. This could be an advantage in conditions of low inotropy but could also lead to relaxation abnormalities and adverse accumulation of Ca^2+^ in cytosol and cell death [106]. On the contrary, the inhibition of NCX in the reverse mode could be of pharmacological importance in limiting the cellular Ca^2+^ content and Ca^2+^ overload in ventricular cardiomyocytes where the NCX activity is increased.

It is evident that remodeling the activity of SERCA2a and RyR2 favors the improvement of Ca^2+^ handling in diabetes. Majority of studies in both models of diabetes have shown that the activity of RyR2 is increased, while the activity of SERCA2a is diminished in diabetic ventricular cardiomyocytes. Suppressing RyR2-mediated SR Ca^2+^ leak by directly modifying RyR2 gating represents an effective strategy for preventing spontaneous Ca^2+^ waves. In this regard, several drugs with unique inhibitory action on Ca^2+^ waves have been tested in earlier studies [107, 108]. These drugs have been shown to possess antiarrhythmic effects and could probably have cardioprotective properties. However, their mechanisms of action are both complex and controversial. Modulation of the RyR2 activity can also be achieved by targeting CaMK II, which inhibits RyR2 phosphorylation and results in an overall decreased SR Ca^2+^ overload [109].

Although the role of RyR2 in excitation-contraction coupling in cardiomyocytes is well established, a functional role for RyR2 in *β* cell insulin secretion is not well understood. Missense mutations in RYR2 were shown to be associated with catecholaminergic polymorphic ventricular tachycardia (CPVT), which is characterized by exercise-induced arrhythmias and sudden cardiac death. CPVT patients were found to have leaky RyR2, present with glucose intolerance. In mice, the transgenic expression of CPVT-associated RyR2 resulted in impaired glucose homeostasis. Furthermore, *β* cells from these animals revealed intracellular Ca^2+^ leak via oxidized and nitrosylated RyR2 channels [110]. It is important to mention that chronic intracellular Ca^2+^ leak via RyR2 channels in the pancreatic *β* cell causes store depletion, triggers ER stress, and results in mitochondrial dysfunction. Consequently, these effects lead to the reduction in ATP synthesis and eventually decreased glucose-stimulated insulin secretion by *β* cells. Impaired mitochondrial function also leads to increased production of ROS, which triggers redox modifications of RyR2, thereby aggravating the Ca^2+^ leak [111]. Therefore, pharmacological inhibition of intracellular Ca^2+^ leak via RyR2 channels in diabetic patients would be critically important.

Many studies in both models of diabetes have shown that the activity of SERCA2a is diminished in diabetic ventricular cardiomyocytes. Therefore, remodeling the activity of SERCA2a would play an important role in improving the process of Ca^2+^ handling in diabetes. The overexpression of SERCA2a and modulation of the inhibitory action of the regulatory protein PLB provide potentially important therapeutic approaches in improving ventricular contractile function in diabetes [12]; however, such approaches will need further extensive studies and testing in relevant animal and preclinical models.

It is worth mentioning that the levels of SERCA can be assayed in peripheral blood lymphocytes, and their levels correlate with SERCA levels obtained in the cardiac tissue [112]. Mechanistically, the decreased SERCA activity results in Ca^2+^ overload in the cytoplasm which is known to be arrhythmogenic. For this reason, assay of SERCA levels could provide valuable information on proarrhythmogenesis. This aspect might help clinicians to identify patients with higher rate of arrhythmic events and worse prognosis. Additionally, SERCA may become a therapeutic target of tailored therapies and interventional approaches to reduce the arrhythmic burden in patients. A recent study was conducted to evaluate atrial fibrillation (AF) recurrence and SERCA levels in patients treated by epicardial thoracoscopic ablation for persistent AF [113]. After a successful epicardial ablation procedure, there was significant increase in the SERCA expression in responders compared to baseline and to nonresponders. Responders also displayed a marked reduction of inflammatory cytokines. The findings of this study indicated that SERCA may represent an effective therapeutic target to reduce postablative recurrences in patients with persistent AF.

## 8. Conclusion

Over the last decade, significant progress has been made in outlining the mechanisms responsible for the altered cardiac contractile function in diabetes using different animal models of TIDM and TIIDM. Exploring the molecular mechanism(s) involved in the disturbances of Ca^2+^ transport and the role of main cardiac proteins responsible for Ca^2+^ homeostasis in the diabetic rat ventricular cardiomyocytes will provide an insight for the identification of novel therapeutic approaches to improve heart function in diabetic patients.

## Figures and Tables

**Table 1:** Effect of DM on the L-type Ca^2+^ channel.

TIDM	Effect	References
	Reduced L-type Ca^2+^ current in STZ-treated rat ventricular myocytes	Hamouda et al. 2015 [23]; Wang et al. 1995 [24]; Chattou et al. 1999 [26]; Bracken et al. 2006 [28]; Woodall et al. 2004 [25]
	Reduced L-type Ca^2+^ current in Akita(ins2) mice	Lu et al. 2007 [27]
	No significant change in the L-type Ca^2+^ current in STZ-treated rat ventricular myocytes	Smail et al. 2016 [19]; Lacombe et al. 2007 [20]; Choi et al. 2002 [21]; Teshima et al. 2000 [22]

TIIDM	Effect	References
	Reduced number of L-type Ca^2+^ channels in sarcolemma in db/db mice	Pereira et al. 2006 [29]
	Reduced density of T-tubular in *db/db* mice	Stølen et al. 2009 [30]
	No change in L-type Ca^2+^ channels in Goto-Kakizaki rats	Salem et al. 2013 [32]; Al Kury et al. 2018 [33]
	Upregulation of the gene encoding Ca_v_1.2 ion channel (Cacna1c)	Howarth et al. 2011 [31]

**Table 2:** Effect of DM on ryanodine receptor type 2.

TIDM	Effect	References
	Decrease in ^3^H-labeled ryanodine binding sites, decrease in the mRNA expression	Yu et al. 1994 [48]; Teshima et al. 2000 [22]; Choi et al. 2002 [21]; Zhao et al. 2014 [49]
	Hyperphosphorylation of RyR2	Yaras et al. 2005 [52]; Shao et al. 2007 [54]; Shao et al. 2009 [51]
	Hyperphosphorylation of RyR2 due to both high phosphorylation levels of both PKA and CaMKII	Tuncay et al. 2014 [53]; Netticadan et al. 2001 [59]
	AGEs on RyR2, disulfide bond formation on RyR2, oxidation of RyR2 by reactive oxygen species (ROS), and/or reactive carbonyl species	Bidasee et al. 2003a [2]; Bidasee et al. 2003b [102]; Shao et al. 2012 [46]
	Slow release of Ca^2+^ from SR and prolonged time to peak Ca^2+^ transients	Choi et al. 2002 [21]

TIIDM	Effect	References
	Decrease in RyR2 mRNA levels in the GK model	Gaber et al. 2014 [62]
	Decrease in [^3^H]ryanodine binding affinity in the right and left ventricle	Dincer et al. 2006 [60]
	Increase in RyR2 phosphorylation at Ser 2808/Ser 2809	Dincer et al. 2006 [60]; Okatan et al. 2016 [61]
	Increased oxidation of RyR2, decreased *S*-nitrosylation, and diastolic Ca^2+^ leak; increased activity in PPAR-*γ* overexpressed mice with high lipid; and increased RyR2 activity due to redox modification in HFD-fed mice	Oda et al. 2015 [66]; Gonzalez et al. 2007 [67]; Joseph et al. 2016 [64]; Xie et al. 2016 [65]; Sánchez et al. 2018 [68]

**Table 3:** Effect of DM on sarcoplasmic reticulum Ca^2+^-ATPase.

TIDM	Effect	References
	Decrease in mRNA level/protein expression of SERCA2a in STZ-treated diabetic rats	Teshima et al. 2000 [22]; Choi et al. 2002 [21]; Kim et al. 2001 [73]; Bidasee et al. 2004 [74]; Lacombe et al. 2007 [20]
	Increase in mRNA level/protein expression of non-phosphorylated PLB in STZ-treated diabetic rats	Choi et al. 2002 [21]; Kim et al. 2001 [73]; Bidasee et al. 2004 [74]
	Decrease in the SERCA2a function in alloxan/STZ-treated diabetic rats	Lopaschuk et al. 1983 [75]; Allo et al. 1991 [76]; Zhao et al. 2014 [49]; Lacombe et al. 2007 [20]
	Inhibition of SERCA2 by free radicals through the direct attack of ATP-binding site	Xu et al. 1997 [78]; Ying et al. 2008 [79]
	Downregulation through posttranslational modifications (glycation, carbonylation, and O-GlcNAcylation)	Bidasee et al. 2004 [74]

TIIDM	Effect	References
	Decreased SR Ca^2+^ uptake, increased PLB phosphorylation, unaltered SERCA2a expression in db/db mouse, and adult rats fed on sucrose	Wold et al. 2005 [80]
	Decreased SERCA2a function, enhanced CaMKII-mediated phosphorylation of PLB in Ob/Ob mice.	Stølen et al. 2009 [30]
	Decreased SERCA2a expression in Zucker Diabetic Fatty rat	Young et al. 2002 [81]
	Increased SERCA2a expression in Zucker Diabetic Fatty rat	Fredersdorf et al. 2012 [82]

**Table 4:** Effect of DM on sodium-calcium exchanger.

TIDM	Effect	References
	Reduction NCX current density	Chattou et al. (1999) [26]; Hattori et al. 2000 [89]; Lacombe et al. 2007 [20]; Sheikh et al. 2012 [90]; Zhao et al. 2014 [49]
	Reduced in NCX mRNA	Hattori et al. 2000 [89]
	Reduced or unaltered NCX protein	Choi et al. 2002 [21]; Lee et al. 2013 [91], Zhang et al. 2013 [92]
	Decreased NCX activity in alloxan-treated rats	Golfman et al. 1998 [93]; Allo et al. 1991 [76]
	Increased NCX expression in Akita(ins2) TIDM	LaRocca et al. 2012 [94]

TIIDM	Effect	References
	No change in the NCX expression and current density in insulin-resistant sucrose-fed rats and HFD mice	Wold et al. 2005 [80]; Ricci et al. 2006 [96]
	Increased activity in the TIIDM model (db/db)	Stølen et al. 2009 [30]
	Increased NCX1 gene expression	Ashrafi et al. 2017 [97]
